# Multi-Omics for Mothers and Infants (MOMI) Consortium: a global initiative to study adverse pregnancy outcomes

**DOI:** 10.7189/jogh.16.05002

**Published:** 2026-04-03

**Authors:** Xin Tang, Salahuddin Ahmed, Farzana Bashir Ahmed, Vincent Albrecht, Vincenth Brennsteiner, Alan Lee Chang, Nabidul Haque Chowdhury, Saikat Deb, Matthew Gormley, Tarik Hasan, Leah Kamulaza, Javairia Khalid, Waqasuddin Khan, Rasheda Khanam, Pallavi Kshetrapal, Hanqi Luo, Tushar K Maiti, Arindam Maitra, Mohammad Mohsin Mannan, Johannes Mueller-Reif, Clyde Mulenga, Imran Nisar, Jesmin Pervin, Teeranan Pokaprakarn, Joni Price, Muhammad F Qazi, Katelyn Rittenhouse, Yuri Sebastiao, Ntazana Sindano, Tulika Sinha, Shailaja Sopory, Medini Steger, Ramachandran Thiruvengadam, Nitya Wadhwa, Lei Xue, Nima Aghaeepour, Abdullah H Baqui, Shinjini Bhatnagar, Susan Fisher, Melissa Fox, Daniela Hampel, Margaret Kasaro, Matthias Mann, Anisur Rahman, Sunil Sazawal, Liat Shenhav, Jeffrey SA Stringer, Ge Zhang, Fyezah Jehan, Kristina De Paris

**Affiliations:** 1 Division of Human Genetics, Cincinnati Children’s Hospital Medical Center, Cincinnati, Ohio, USA; 2 Projahnmo Research Foundation, Dhaka, Bangladesh; 3 Section of Pediatric Infectious Diseases, Department of Pediatrics and Child Health, Aga Khan University, Karachi, Pakistan; 4 Research Department of Proteomics and Signal Transduction, Max Planck Institute of Biochemistry, Martinsried, Germany; 5 Department of Anesthesiology, Perioperative and Pain Medicine, Stanford University, Palo Alto, California, USA; 6 Public Health Laboratory and Center for Public Health Kinetics, Pemba, Zanzibar, Tanzania; 7 Department of International Health, International Center for Maternal and Newborn Health, Bloomberg School of Public Health, Johns Hopkins University, Baltimore, Maryland, USA; 8 Department of Obstetrics, Gynecology, and Reproductive Sciences, University of California, San Francisco, California, USA; 9 University of Zambia, UNC Global Zambia, Lusaka, Zambia; 10 Translational Health Science and Technology Institute, Faridabad, India; 11 Department of Global Health, Rollins School of Public Health, Emory University, Atlanta, Georgia, USA; 12 Institute of Basic and Biomedical Sciences, Levy Mwanawasa Medical University, Lusaka, Zambia; 13 Maternal and Child Health Division, International Centre for Diarrhoeal Disease Research, Maternal, Newborn, and Child Health, Dhaka, Bangladesh; 14 Division of Global Women’s Health, School of Medicine, University of North Carolina, Chapel Hill, North Carolina, USA; 15 Department of Biomedical Data Science, Stanford University, Palo Alto, California, USA; 16 Department of Nutrition, University of California, Davis, California, USA; 17 Institute for Systems Genetics, Department of Microbiology, Department of Obstetrics and Gynecology, Grossman School of Medicine, New York University, New York, New York, USA; 18 Department of Microbiology and Immunology, School of Medicine, Chapel Hill, University of North Carolina, North Carolina, USA

## Abstract

**Background:**

The Multi-Omics for Mothers and Infants (MOMI) Consortium aims to define biological mechanisms associated with preterm birth, small for gestational age, preeclampsia, and stillbirth. Globally, the burden of adverse pregnancy outcomes (APOs) is highest in low- and middle-income countries (LMICs). The MOMI Consortium consists of six different LMIC sites established by The Alliance for Maternal and Newborn Health Improvement in Bangladesh, Pakistan, and Tanzania, the Global Alliance to Prevent Prematurity and Stillbirth in Bangladesh and Zambia, and the Interdisciplinary Group for Advanced Research on Birth Outcomes – DBT India Initiative. It also partners with five analytical partners and three bioinformatic teams.

**Methods:**

The combined MOMI cohort includes 24 321 pregnant women, a rich biorepository, and paired socioeconomic and clinical data. Considering the multifactorial aetiologies and molecular drivers of APOs, we applied an integrative multi-omics (genomics, metabolomics, proteomics, nutrient testing) approach to identify site-specific and cohort-wide signatures linked to distinct APOs.

**Conclusions:**

This protocol summarises the MOMI study design, sample and data collection methods, data harmonisation, and the various analytics platforms, and discusses potential outcomes for enhanced clinical care and novel diagnostic and therapeutic tools.

Pregnancy complications such as preeclampsia (PE) and adverse pregnancy outcomes (APOs) such as preterm birth (PTB), small for gestational age (SGA), and stillbirth (SB) pose a serious global health challenge and collectively contribute to high rates of maternal and infant mortality and morbidity. Close to 2 million stillbirths were reported in 2020 [1,2] while more than half (55.3%) of the 2.4 million neonatal deaths were associated with PTB and/or SGA [1,2]. While numerous studies have explored the biological mechanisms of APOs, small sample size, geographic limitations, and inconsistent study designs hinder the generalisability of the findings. For example, a recent review of 147 studies on 56 pregnancy and pre-pregnancy cohorts from 26 different countries demonstrated that most studies were conducted in high-income countries, while specimens were collected in only about half of the studies and at varying time points [3].

Comprehensive multi-cohort studies with harmonised clinical data and longitudinally collected biological specimens at time points standardised across sites and cohorts are required to address these challenges. The Multi-omics for Mothers and Infants (MOMI) Consortium was formed in response to this need. It brings together diverse pregnancy cohorts from low- and middle-income countries (LMICs) in Asia and Africa: the Alliance for Maternal and Newborn Health Improvement (AMANHI) from Pakistan, Bangladesh, Tanzania; the Global Alliance to Prevent Prematurity and Stillbirth (GAPPS) from Bangladesh and Zambia; and the Interdisciplinary Group for Advanced Research on Birth Outcomes – DBT India Initiative (GARBH-INi). This creates a unique resource of 24 321 pregnant women with well-phenotyped pregnancy outcomes and longitudinally collected biospecimens. The Consortium partnered with five analytics partners and three bioinformatics teams to analyse longitudinally collected specimens for genomics (Cincinnati Children’s Hospital, Ohio, USA), metabolomics (Sapient Bio, California, USA), proteomics (Max Planck Institute for Biochemistry, Martinsried, Germany), and nutrient testing (UC Davis, California, USA, and International Centre for Diarrhoeal Disease Research (icddr,b), Dhaka, Bangladesh). The vast dataset of the various multi-omics and nutrient analyses, in combination with the sociodemographic and clinical metadata, will enable the MOMI Consortium to identify causal pathways through data integration and validate predictive biomarkers to develop clinically relevant interventions for mitigating APOs.

Only a few other studies of similar scope and magnitude have been performed or are ongoing. Analogous to the MOMI study design, a prospective study in China follows approximately 20 000 women in rural and urban centres to document maternal pregnancy outcomes and infant health [4]. The aforementioned Chinese study also aims to establish a large biorepository that will be utilised to define mechanisms associated with adverse outcomes among mothers and children by applying various omics platforms. The potential usefulness of such a multi-omics approaches in identifying biological pathways promoting APOs has been highlighted in a review about PE [5,6]. There are several other large-scale country-specific initiatives, such as the ORIGNS pregnancy and birth cohort in Western Australia [7], or the All of US Pregnancy cohort and the Nulliparous Pregnancy outcomes Study: Monitoring Mothers-To-Be in the US [8,9]. Most of these studies have established large biorepositories and databanks that are now accessible to researchers. Examples include IMPROVed [10], a biobank to study PE utilising specimens from four different European countries, or the Collaborative Online Perinatal and Pediatric Repository [11]. Precedence for the potential success of the international MOMI Consortium has been provided by other large global networks, such as the Intergrowth 21st project and its extension, the INTERBIO-21st project. The Intergrowth 21st project was an international collaborative consortium to establish international standards for foetal growth and to determine the association between gestational weight gain and APOs. The INTERBIO-21 is an extension of the Intergrowth 21 project that intends to define the impact of APOs on infant morbidity up to two years of age [6,12-14]. Akin to the Intergrowth 21st Network, the MOMI Consortium represents an international collaboration of clinicians and researchers in LMICs that were specifically chosen for their high incidence of APOs.

We hypothesise that the integration of data from multiple omics technologies will provide the means to define biological pathways that are altered in pregnant women with APOs and that, despite some overlap, unique signatures for PTB, PE, stillbirth, and SGA can be deduced to develop novel risk assessment tools and interventions. The inherent heterogeneity across the cohorts in different geographical locations will be counterbalanced by the tightly coordinated study design, data harmonisation, and adjudication of birth outcomes, and the extensively validated computational pipeline developed in prior studies [15-21]. Furthermore, only APO signatures that are reproducible across different cohort sites will be considered biologically and clinically meaningful. This protocol presents the objectives, study design, and analytical approaches of the MOMI Consortium and outlines how integration of multi-omics and metadata will enhance the understanding of pregnancy biology, inform risk prediction models, and contribute to the development of targeted interventions to improve maternal and infant health.

## STUDY DESIGN AND CONSORTIUM STRUCTURE

### Goals and governance

**Figure 1:**
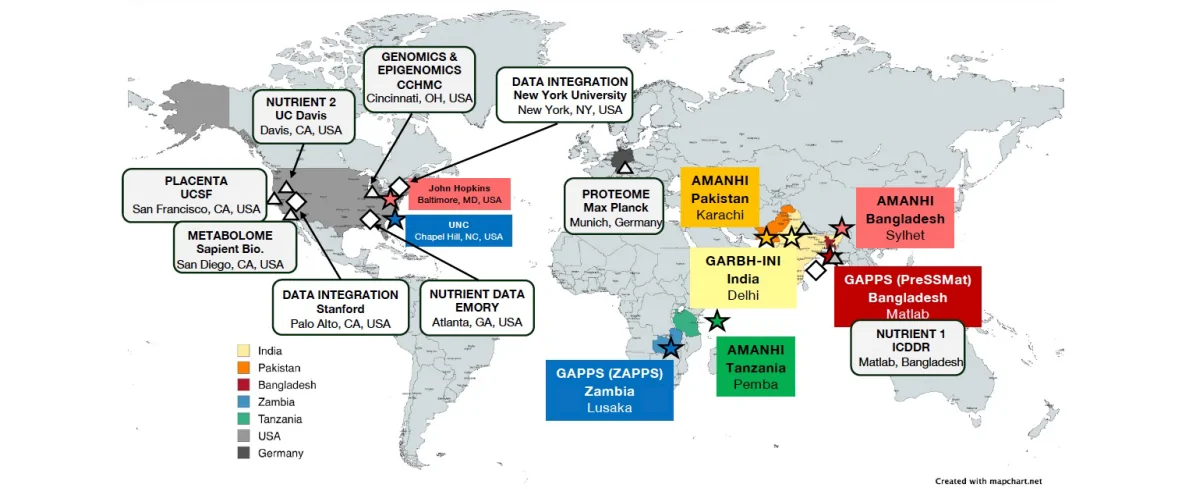
MOMI Consortium structure. The world map lists the five countries in Southeast Asia and Africa with their pregnancy cohort sites (star symbol), with colour-coded boxes listing the specific study initiatives and the corresponding country and city. Collaborating US university partners for AMANHI Bangladesh and GAPPS Zambia are indicated by their relevant colour-matched stars and boxes. Note that the MOMI cohort consists of the AMANHI and GAPPS cohorts, with the GARBH-INI cohort study being separate. Triangles and grey boxes name the sites of omics analytics partners. Data coordination and integration sites are indicated by white diamonds and text boxes. Source and permission to use: Created maps, licensed under Creative Commons Attribution – ShareAlike 4.0 International License. AKU – Aga Khan University, Pakistan, BIRAC – Biotechnology Industry Research Assistance Council, India, CCHMC – Cincinnati Children's Hospital Medical Center, USA, CPHK – Center for Public Health Kinetics, Tanzania, icddr,b – International Centre for Diarrhoeal Disease Research, Bangladesh, JHU – Johns Hopkins University, USA, MPI – Max Planck Institute of Biochemistry in Martinsried, Germany, Sapient – Sapient Bioanalytic LLC, San Diego, USA, THSTI – Translational Health Science and Technology Institute, India, UNC – University of North Carolina at Chapel Hill, USA, USDA/UCD – United States Department of Agriculture/University of California at Davis, USA.

**Figure 2:**
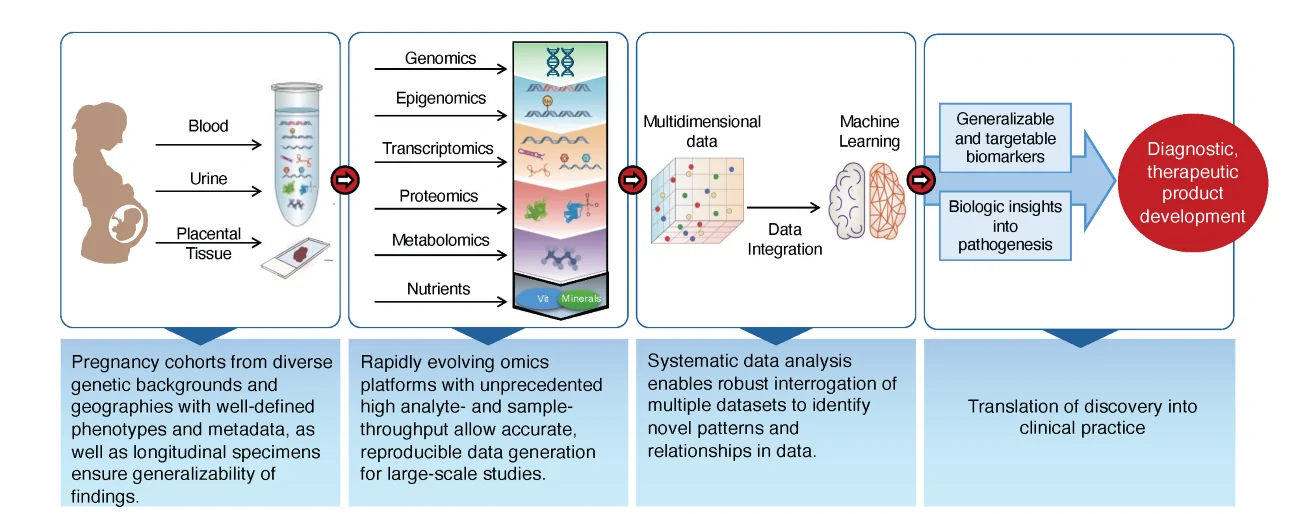
Study flow and overview.

The MOMI Consortium (**Figure 1**) was established in 2018 to leverage large pregnancy cohorts in representative Asian and African LMICs with high risk for adverse pregnancy outcomes. Applying a set of distinct omics technologies to longitudinally collected specimen from women with term birth versus women with adverse pregnancy outcomes combined with coordinated data analysis and integration at each site and across sites was key to achieving the MOMI goals (**Figure 2**). The Consortium agreement provides a structured ethical framework, outlining data sharing, intellectual property rights, and authorship policies to ensure equitable contributions from all partners and emphasising fair collaboration between Global North and South institutions (Figure S1 in the **Online Supplementary Document**).

### Cohorts and specimen collection

The MOMI sites (**Figure 1**) established prospective pregnancy cohorts incorporating longitudinal clinical visits and specimen collection at time points strategically chosen to represent specific gestational age (GA) windows within each pregnancy trimester (**Table 1**). While the GAPPS and AMANHI studies were primarily community-based studies, the GARBH-INi study was hospital-based. Pregnant women were enrolled at <20 weeks of GA at all sites, except Zambia, where women were enrolled up to 24 weeks GA because 44.6% of the GAPPS Zambia cohort represented women living with HIV who often present later to antenatal care. The total MOMI cohort encompassed 16 221 pregnant women from the AMANHI and GAPPS studies, while GARBH-INi consisted of 8100 women. Each site followed standardised protocols for specimen collection and storage, ensuring comparability across sites. Specimens included maternal urine, plasma, serum, whole blood, buffy coats, placenta tissue (basal plate, villous chorion and chorionic plate), placenta membrane, foetal cord blood, neonatal saliva, infant blood, and faecal samples [16,17,19,22-24]. Urine, serum, and plasma were stored at −80°C. Ethylenediaminetetraacetic acid-anticoagulated whole blood was stored frozen at −80°C, preserved as dried blood spots, or processed into buffy coats and stored at the same temperature. Placental tissues were preserved in RNAlater, formalin, or snap frozen. Each site established its own biorepository with specimen tracking through laboratory data management systems, with the biorepositories in Bangladesh, Pakistan, and Tanzania being the first pregnancy-related biobanks in LMICs [22].

**Table 1:** Study cohorts and specimen collection

	Cohort					
**Cohort initiative**	**GAPPS**		**AMANHI**			**GARBH-INI**
**Country**	**Zambia (ZAPPS)**	**Bangladesh (PreSSMat)**	**Bangladesh**	**Pakistan**	**Tanzania**	**India**
**City**	Lusaka	Matlab	Sylhet	Karachi	Pemba	Delhi
**Study time**	August 2025 to August 2021	August 2015 to August 2017	May 2014 to June 2018	May 2014 to June 2018	May 2014 to June 2018	May 2015, ongoing
**Cohort size**	2576	3644	3000	2500	4501	8100
**Study visits**						
Enrollment	<24 weeks GA*	<20 weeks GA	<20 weeks GA			<20 weeks GA
Post enrollment, during pregnancy						10–14 weeks GA
	22–24 weeks GA		24–28 weeks GA			18–20 weeks GA
	26–28 weeks GA					26–28 weeks GA
	32 weeks GA		32–36 weeks GA			30–32 weeks GA
	36 weeks GA		Post-37 weeks GA			No
Delivery	Yes		Yes			Yes
Post-delivery	Week 6		1–6 days and infant at 42–60 days			Six weeks to six months
**Maternal specimens**						
Urine	Yes	Yes	Yes	Yes	Yes	Yes
Serum	Yes	Yes	Yes	Yes	Yes	Yes
Plasma	Yes	Yes	Yes	Yes	Yes	Yes
Blood	Buffy coat, whole blood	Whole blood	Buffy coat, DBS	Buffy coat	Buffy coat	DNA of whole blood
Placenta tissue	RNAlater, formalin, snap frozen		RNAlater, formalin, snap frozen			Formalin, snap frozen
Placenta membrane	RNAlater, formalin, snap frozen		RNAlater, formalin, snap frozen	No	RNAlater, formalin, snap frozen	RNAlater, formalin, snap frozen
Faecal sample (delivery)	Yes	No	Yes	Yes	No	No
**Infant specimens**						
Blood	Cord blood, DBS	Cord blood	DBS (72 hours)	Cord blood buffy coat	Cord blood	DNA of cord blood
Faecal Sample	Yes (delivery)	No	Yes (42–60 days)	Yes (42–60 days)	Yes (42–60 days)	No

AMANHI – Alliance for Maternal and Newborn Health Improvement, GAPPS – Global Alliance to Prevent Prematurity and Stillbirth, GARBH-INI – Interdisciplinary Group for Advanced Research on Birth Outcomes – DBT India Initiative, DBS – dried blood spots, GA – gestational age, PreSSMat – Preterm and Stillbirth Study, Matlab, ZAPPS – Zambian Preterm Birth Prevention Study

*Women living without HIV: 20 weeks of GA; women living with HIV: <24 weeks of GA.

### Sociodemographic and clinical data

Sociodemographic data (age, partner status, education, tobacco, alcohol and drug use), weight, medical history, parity status and, in case of prior pregnancy, pregnancy history and outcome, were documented at enrolment. A medical exam was conducted at the same time point that included body mass index, blood pressure (BP), and haemoglobin measurement, urine analysis, testing for HIV and other sexually transmitted diseases, assessment of pregnancy symptoms, and a dating ultrasound. Subsequent antenatal visits included determination of GA, recording of pregnancy symptoms since the prior visit, and testing for urine, haemoglobin, BP, heart rate, and weight. At delivery, medical conditions or medications during pregnancy and/or delivery, time of membrane rupture, delivery time, and birth outcome were documented. The foetus’s heart rate and fundal height were also assessed. Data also captured indications for provider-initiated delivery or caesarean section, postpartum complications, neonatal vital status, and Apgar score. Women and infants were seen at least once between six weeks to six months post-delivery. The infant was evaluated for any diseases and medications since birth, and evaluated for infant weight, head circumference, mid-upper arm circumference, temperature, heart and respiratory rates. The mother underwent a pelvic exam and was assessed for weight, BP, heart rate, temperature, and oedema.

### Study design

**Figure 3:**
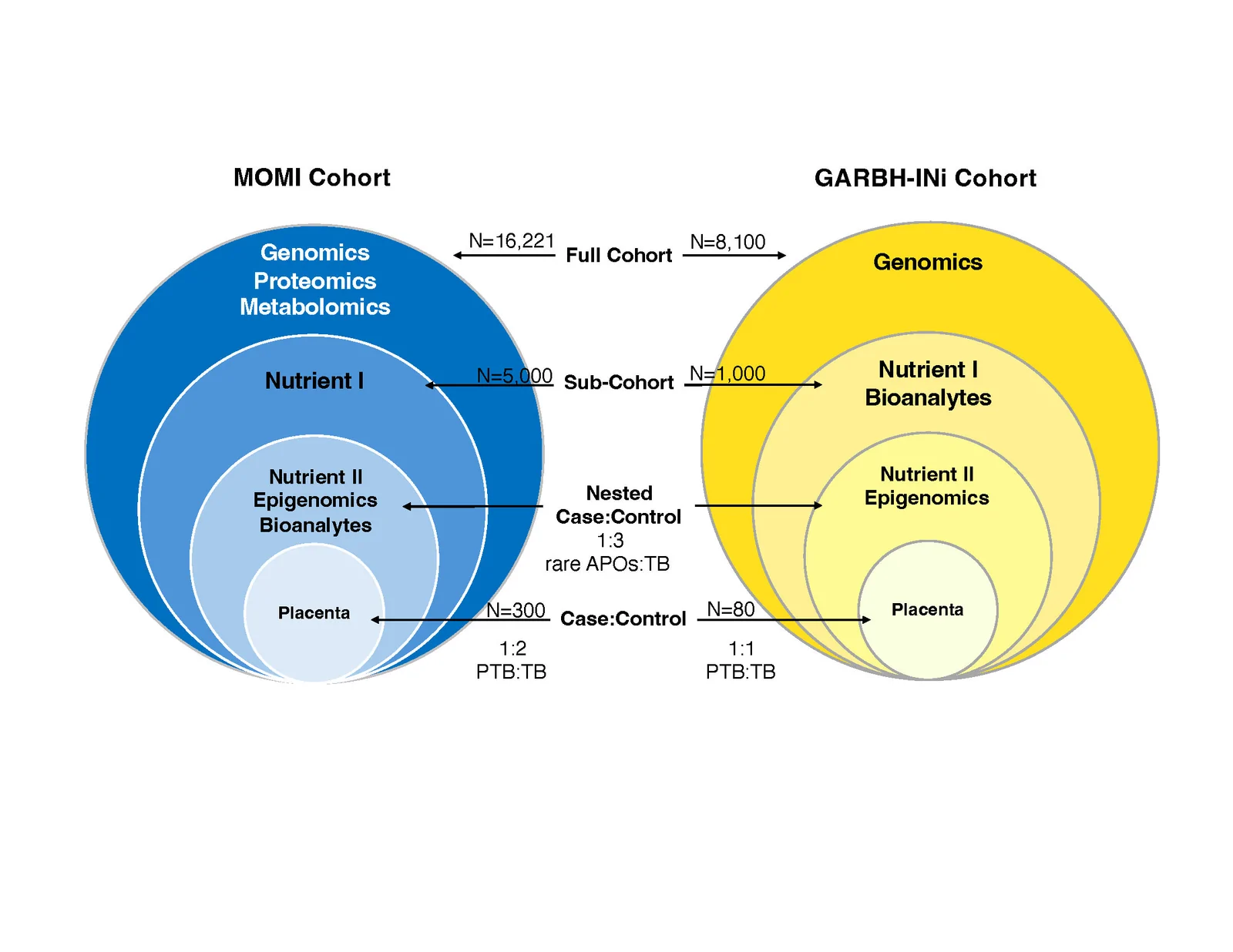
Study design**.** The general design for the multi-omics analysis was similar in both the MOMI cohort sites and the GARBH-INI cohort, with slight differences in specimen numbers assigned to the various analyses. Genomics testing was performed on the full cohort using maternal longitudinal blood samples and infant cord blood. Specimens of the full MOMI cohort were also analysed for plasma proteome and metabolome. A sub-cohort study was conducted with ~30% of MOMI cohort women and maternal specimens from 1000 women of the GARBH-INI cohort to test longitudinal serum and/or urine specimens for nutrients group I. In the GARBH-INI cohort, additional testing for common bioanalytes (TSH, PAPP-A, sFlt, PlGF) was performed using plasma. The sub-cohort study included all cases of common (*e.g*. PTB, SGA) and rare APOs (very PTB, PE). In the GARBH-INI cohort, specimens of the sub-cohort study will also be tested for proteomic and metabolomic signatures identified in the full MOMI cohort. An additional nested cohort study was conducted using all rare APOs (very PTB, PE, SB) at a 1:3 ratio to term (TB) birth outcomes. Specimens in the nested cohort study were analysed for nutrients group II and Epigenomics, and, in the MOMI cohort only, for common bioanalytes. Finally, placental tissues from women of the GAPPS and AMANHI sites with preterm and term births are undergoing histopathology (154 and 323 samples, respectively), transcriptomic (26 and 56 samples, respectively), single nuclei RNAseq and ATACseq (15 and 30 samples, respectively), and laser capture/mass spectrometry proteomic analysis (32 and 62 samples, respectively). In the GARBH-INI cohort n=40 women with PTB will be analysed for histopathology and a subset of these will be analysed for transcriptomic markers in comparison to TB (1:1). Please see text for further study details.

The MOMI Consortium progressed through three phases. The pre-pilot phase focused on sample quality assessment. The pilot phase was dedicated to the development and validation of the analytical workflows, while simultaneously harmonising the MOMI dataset, study outcomes, and analytical approaches [16,17,19,23,24]. The current phase (**Figure 3**) implemented population-scale molecular profiling of relevant specimens and continues with ongoing site-specific and cohort-wide data analysis and integration. Analyses are being performed on specimens collected during early (enrolment at <20 or <24 weeks GA), mid (22–28 weeks GA), and late pregnancy (28–36 weeks GA). These time points were selected to determine the dynamic trajectory of analytical factors throughout pregnancy, and test whether specific molecular signatures at one or multiple time points can predict the risk for specific APOs. Clinically, the earlier a risk for a specific APO can be identified, the higher the potential for adequate surveillance, improved clinical care, and prevention or reduced severity of the APO. Among the omics-platforms applied, metabolomics and proteomics will be applied to all longitudinal maternal specimens and cord blood, while the genomics analysis requires only a single sample from mother and infant. Maternal GARBH-INi specimens will also undergo genomics analysis. A sub-cohort study will include the longitudinal specimens of women with the most common APOs (PTB and SGA) and rare APOs (SB, very PTB, PE) plus a random sample from the full cohort (n = 5000) and 1000 women from the GARBH-INi cohort. These specimens are being analysed for nutrients group I, while in the GARBH-INi cohort, they are also being investigated for common bioanalytes and for proteomic and metabolomic APO signatures identified in the MOMI cohort. A nested case-control study (1:3) of the sub-cohort will consist of all specimens from women with rare APOs compared to women with term birth to test for biomarkers of epigenomics, nutrients of group II, and common bioanalytes. The common bioanalytes were selected based on their association with specific APOs and will only be measured at time points relevant to their role in pregnancy. Specifically, thyroid-stimulating hormone and pregnancy-associated plasma protein A will be tested in early pregnancy, whereas soluble fms-like tyrosine kinase and placental growth factor will be tested at mid and late pregnancy [25-31]. Placental tissues from a subset of women with PTB or term birth will undergo histopathology, transcriptomic, epigenomic, and global proteomic analysis.

### Statistical considerations

The case-cohort design was selected to balance statistical power, feasibility, and cost in a large, globally distributed pregnancy cohort. Full multi-omic profiling of all participants is neither economically nor logistically feasible, particularly for low-prevalence outcomes such as stillbirth and PE. The case-cohort framework enables comprehensive inclusion of all cases for each adverse pregnancy outcome while sampling a representative sub-cohort from the full population. This preserves the ability to estimate population-level risks and outcome prevalence, while substantially reducing assay volume and cost. This design also supports both outcome-specific analyses and integrative cross-outcome comparisons through the use of a shared reference sub-cohort. It retains the inferential strengths of a prospective cohort, while enabling deep molecular characterisation of rare and clinically important phenotypes. When integrating molecular and clinical data into cohort-level analyses, we will apply established case-cohort weighting and variance estimation methods (*e.g.* Prentice-weighted Cox models and survey-weighted regression frameworks) to ensure unbiased population-level inference and correct standard errors.

Nested case-control sampling is used for targeted assays and discovery-oriented analyses where relative contrasts between cases and matched controls are most informative and cost-efficient. These analyses will be interpreted as conditional, outcome-specific comparisons and will not be pooled directly with population-level estimates derived from the case-cohort framework. Where results from nested case-control analyses inform broader models, they will be re-estimated within the weighted case-cohort structure to preserve representativeness and avoid selection bias.

### Statistical power

For high-dimensional, regularised machine-learning models operating across multiple molecular modalities, classical closed-form power calculations are not available. Instead, effective power is governed by the number of outcome events, feature dimensionality, and the cross-validation and regularisation strategy. The MOMI substantially exceeds the scale of prior pregnancy multi-omics studies, including those that have successfully identified reproducible molecular signatures of PE and PTB [17,19,20]. The inclusion of all cases for each adverse outcome, coupled with a large, representative sub-cohort, maximises event density and information content, while maintaining cohort-level validity.

## DATA HARMONISATION

The Consortium has developed a harmonised framework for sociodemographic, clinical, and phenotypic variables across the biorepository sites. Specifically, SAS/STATA programmes were developed for harmonising site-specific data sets and quality control programmes for ensuring data consistency, while all harmonised datasets will be uploaded to a centralised platform. The biorepository sites upload clinical and phenotypic metadata that can be accessed by the analytical partners, who, conversely, upload their respective analytical data for cohort investigators. Although the GARBH-INi data, in compliance with government regulations in India and the GARBH-INi funding structure, are controlled by their team, data and analyses pipelines have been harmonised with the overarching MOMI Consortium. A first critical step was the harmonisation of birth outcome data, first on a site-specific level and then through central adjudication for confirmation.

PTB was defined as babies born alive before 37 weeks of pregnancy are completed [32]. Three subcategories were defined based on the GA: extremely preterm (<28 weeks, 0 days GA), (very preterm (28 weeks, 0 days to 31 weeks, 6 days GA), and moderate to late preterm (32 weeks, 0 days to 36 weeks, 6 days GA). The GA assessment was based on the Hadlock formula, which was implementable across all sites; missing head circumference data at AMANHI sites prevented the implementation of INTERGROWTH-21st formula. At time points with available ultrasound data, the GA was computed using either crown rump length or a composite of femur length and biparietal diameter. For the purpose of this study, preterm stillbirths will be evaluated as a part of SB outcomes.

SGA was defined based on the INTERGROWTH-21st standards [33] using the 10th percentile as cut-off. Only some cohort sites were able to define fifth or third percentile SGA babies. Due to discrepancies in the measurement of birth weight among the different cohort sites, birthweights were coded as level 1 or 2 when birthweights were measured within 48 or 72 hours of delivery, respectively, and as level 3 when measured at three days or later.

SB was defined as delivery of a foetus at or after 22 weeks of gestation with no signs of life upon delivery. Among total SB cases, we classified early SB as birth prior to or at 22 weeks, 0 days to 27 weeks, 6 days of GA, and late SB when it occurred at 28 weeks, 0 days GA or later. Otherwise, SB of PTB babies were included in SB case numbers. Clinical data were used to adjudicate antepartum SB (Figure S2 in the **Online Supplementary Document**). Antepartum SB was considered priority 1 when cardiotocographic or ultrasound data of foetal death before onset of labour or rupture of membranes were available. Evidence of maceration, mummification, or other signs of putrefaction of the stillborn foetus was ascribed as priority 2 antepartum SB. An absence of perceived foetal movements as reported by the mother before the onset of labour or rupture of membranes was termed priority 3 antepartum SB.

PE harmonisation was the most complex adjudication process (Figure S3 in the **Online Supplementary Document**). Because pre-pregnancy BP measurements and antihypertensive histories were not systematically available across sites, chronic hypertension could not be uniformly excluded at enrolment. To preserve phenotype specificity under these constraints, PE classification relied on a conservative adjudication framework incorporating evidence beyond elevated BP alone, incorporating maternal end organ dysfunction, proteinuria, and foetal growth restriction occurring after 20 weeks’ gestation. Women with BP 140 mmHg systolic or 90 mmHg diastolic at least four hours apart or at two visits and accompanied by proteinuria at the time of assessment were classified as PE. Women with severe BPs (BP 160 mmHg systolic or 110 mmHg diastolic) were classified as PE regardless of proteinuria. Women with proteinuria documented at a visit not coinciding with elevated BP were classified as PE if urinary tract infection was not suspected and end organ dysfunction was present. Maternal end organ dysfunctions considered for PE diagnosis include haematological dysfunction (*e.g.* thrombocytopenia), renal insufficiency, impaired liver function, pulmonary oedema, cerebral/visual symptoms (*e.g.* headaches, scotomata), and ultrasound-based diagnosed foetal growth restriction or birth weight <3 percentile based on GA. In the absence of proteinuria, women with non-severe BP and documented chronic pre-existing hypertension <20 weeks GA were excluded as PE cases. Women without proteinuria were classified as PE if they had severe BP (>160 mm systolic and/or >110 mm diastolic), without consideration of pre-existing hypertension status; had no pre-existing hypertension <20 weeks but presented with indications of end organ dysfunction >20 weeks; or experienced seizure >20 weeks without history of epilepsy (eclampsia). Based on these criteria, four categories of PE cases were defined, ranging from priority 1 (highest confidence) to priority 3 (lowest confidence), with remaining cases classified as non-PE. Once PE cases were confirmed by central adjudication, priority 1 and 2 PE cases were further stratified into early-onset PE and late-onset PE. The former was defined as PE developing before 34 weeks 6 days GA, and the latter as PE developing at or after 35 weeks.

### Frequencies of APOs

After applying the data harmonisation results, we found that approximately 34% of all pregnancies in the MOMI cohort were affected by adverse outcomes (**Table 2**). Pregnant women in Tanzania experienced the fewest number of APOs (18.33%), whereas pregnancies in women of the other two AMANHI sites (Bangladesh and Pakistan) resulted in the highest numbers of APOs (47.94% and 45.00%, respectively). SGA was the most common APO in the combined MOMI cohort (n = 3527, 21.74% of pregnancies), as well as at each site. PTB was observed in close to 10% of all pregnancies, ranging from 5.4% in Tanzania to 14.9% in Pakistan. PE and SB cases ranged from 0.23% to 3.30% and from 0.64% to 2.41%, respectively. The frequencies of the distinct APOs in the GARBH-INi cohort mirrored those observed in the MOMI cohort (**Table 2**).

**Table 2:** Frequencies of adverse pregnancy outcomes

	GAPPS		AMANHI			Combined MOMI cohort	GARBH-INi
	**Zambia**	**Bangladesh**	**Bangladesh**	**Pakistan**	**Tanzania**		
**Number of Pregnancies**	2576	3644	3000	2500	4501	16 221	8100
**APO**	**Number of APOs (% of pregnancies)**						
**PTB**	223 (8.66)	384 (10.54)	366 (12.20)	372 (14.88)	242 (5.38)	1587 (9.78)	764 (9.43)
Very PTB (% PTB)*	45 (20.0)	14 (3.6)	40 (10.9)	20 (5.4)	29 (11.9)	148	72 (9.4)
**PE**	85 (3.30)	35 (0.96)	7 (0.23)	39 (1.56)	124^c^(2.75)	290 (1.79)	166 (2.05)
Early-onset PE (% PE)	35 (41.2)	7 (20.0)	3 (42.8)	9 (23.1)	22 (17.7)	76	13 (8.6)
Late-onset PE (% PE)	50 (58.8)	28 (80.0)	4 (57.2)	30 (76.9)	100 (80.6)	212	151 (91.0)
**SGA**							
SGA <10th percentile	434 (16.84)	936 (25.68)	1044 (34.80)	683 (27.32)	430 (9.55)	3527 (21.74)	2402 (29.65)
SGA <3rd percentile	167	385	525	304	170	1551	1167
**Stillbirth**	62 (2.41)	25 (0.69)	21 (0.70)	31 (1.24)	29 (0.64)	168 (1.04)	168 (2.07)
**Total number of APOs**	804 (31.21)	1380 (37.87)	1438 (47.94)	1125 (45.00)	825 (18.33)	5572 (34.35)	3500 (43.20)

AMANHI – Alliance for Maternal and Newborn Health Improvement, APO – adverse pregnancy outcome, GAPPS – Global Alliance to Prevent Prematurity and Stillbirth, MOMI – Multi-Omics for Mothers and Infants, PE – preeclampsia, PTB – preterm birth, SGA – small for gestational age

*Very PTB is defined as birth <31 weeks/6 days gestational age.

†Due to missing GA at birth, early-onset and late-onset PE cannot be derived for n = 2.

Thus, we have established a unique two-continent spanning cohort of pregnant women in LMICs, of which more than a third experienced APOs. Thousands of longitudinally collected specimens are now being analysed for genetic, proteomic, metabolomic, or nutrient factors associated with specific pregnancy outcomes. According to the study design (**Figure 3**), specimens from a single time point were selected for genomics analysis, whereas proteomic and metabolomic signatures will be determined using over 30 000 longitudinally collected maternal plasma samples (Table S1 in the **Online Supplementary Document**). Nutrients type I will be measured in >9500 maternal plasma and urine samples, and approximately 1200 maternal plasma samples for nutrients type II (Table S1 in the **Online Supplementary Document**). Specimen numbers for placental tissue analysis are listed in the relevant section below. To gain insights into potential biological factors that may impact infant morbidity and mortality and, considering the complex interactions of the mother-infant dyad, the same analytical tools are being applied to cord blood samples (Table S1 in the **Online Supplementary Document**).

## ANALYTICAL STUDIES

Genomics have emerged as a powerful tool to identify genetic risk factors, both maternal and foetal, of APOs [34-37]. As the association of genetic factors with certain outcomes generally requires a large sample size, the genomics analysis is performed for the full cohort to understand the genetic basis of pregnancy phenotypes and the variation of other omic features. The core steps include the generation of genomic data of maternal and foetal DNA samples using 1 × low-pass whole-genome sequencing, followed by high-quality imputations [38] using large reference panels [39]; conducting genomic analyses of multiple pregnancy phenotypes in mother/infant pairs collected from diverse populations; and integration with other omics measures. Given the close maternal-foetal interaction, pregnancy phenotypes are influenced by both genomes. Therefore, in addition to conventional genomic analysis in mothers and infants separately, the aforementioned genomic analysis will also be conducted in mother/child pairs using a haplotyped-based approach [25], in which a mother-child pair will be treated as the analytical unit with three haplotypes each with distinct maternal or foetal genetic effects. The haplotype-based genomic data analysis will be conducted at three different levels: genome-wide scan for single-variant associations; multi-variant analysis to examine the cumulative effects of multiple variants; and analysis of genome-wide variants to study the genetic architecture (Figure S4 in the **Online Supplementary Document**).

Epigenomics plays a vital role in understanding how genes interact with environmental factors during pregnancy, significantly influencing maternal and foetal health [40-42]. The MOMI study addresses two key questions in human pregnancy: the association between maternal epigenetic marks and pregnancy outcomes, and foetal epigenetic alterations resulting from suboptimal in utero exposures. The epigenomic analysis will be conducted on a selected subset of approximately 1500 pregnancies, enriched for cases of very PTB, PE, and SB (**Figure 3**). Genome-wide methylation profiling will be performed using the Infinium MethylationEPIC v2.0 BeadChip (Illumina Inc., San Diego, California, USA) on maternal DNA samples extracted from whole blood or buffy coats collected at enrolment, as well as foetal DNA extracted from cord blood or buffy coats (Figure S5 in the **Online Supplementary Document**). Future studies will analyse maternal blood samples collected during follow-up visits to investigate dynamic methylation changes throughout pregnancy. The proposed analytical aims include: testing maternal and foetal DNA methylation associations with pregnancy phenotypes and exposures; developing predictive models for APOs; interrogating methylation associations with maternal exposures, micronutrients, other omics measures; methylation quantitative trait loci (mQTL) analysis and causal inferences by Mendelian randomisation (MR). Although the current epigenomic subset presents power constraints, we will ensure the robustness of our causal inference by restricting analyses to cis-acting mQTLs with high instrumental strength (*e.g.* F-statistics >20) and accounting for ancestry heterogeneity using genetic principal components and stratified sensitivity analyses. With approximately 1500 samples, the study is adequately powered to detect cis-mQTLs explaining approximately 2–5% of methylation variance at stringent genome-wide significance thresholds (α ≈ 5 × 10^−8^). For phenotypic outcomes and MR analyses, this sample size allows for the detection of effects explaining approximately 0.6% of variance at a nominal significance level. To maximise discovery power, we supplement single-site MR with multi-variant approaches, including methylation risk scores and polygenic scores. These analyses are framed as supportive and hypothesis-generating, providing a methodological foundation for future consortium efforts that will expand epigenomic profiling to substantially larger samples.

Proteomics alone or in combination with other omics platform have been successfully applied to clinical phenotypes [43-46]. In the MOMI Consortium, the maternal plasma proteome will be defined across the whole pregnancy (Figure S6 in the **Online Supplementary Document**). The study is built upon perfloric acid workflow with neutralisation, an optimised, high-throughput plasma preparation workflow based on perchloric acid precipitation, which significantly reduces costs with great proteomic depth [47]. Samples are analysed using next-generation Thermo Orbitrap Astral (ThermoFisher Scientific, Rheinfelden, Germany) mass spectrometers, providing increased sensitivity and scanning speed essential for deep proteome analysis. To maximise statistical power for biomarker discovery, the study follows a ‘rectangular’ study design [48]. The proteomics analytical pipeline integrates both established and novel computational approaches, including the recently developed AlphaDIA software [49] and proceeds systematically starting from individual cohorts advancing to cross-cohort integration. Quality control measures include standardised reference samples and replicate analyses of pooled samples within each preparation batch. The computational pipeline employs dimensionality reduction and unsupervised clustering for sample classification, followed by statistical modelling to identify proteins associated with APOs, while accounting for confounding factors. Longitudinal analysis will map protein trajectories throughout normal pregnancy progression, providing critical context for identifying pathological deviations. Statistical approaches will be complemented by machine learning to identify predictive protein panels, facilitating the development of diagnostic and prognostic assays and prevention of APOs [50]. This multi-layered analytical strategy is designed to distinguish universal molecular signatures from population-specific variations, advancing our understanding of proteomics changes in pregnancy and their implications for maternal and foetal health.

The metabolome will be analysed by rapid liquid chromatography-mass spectrometry that performs nontargeted measurement of >11 000 small molecule biomarkers, ranging from very polar to very nonpolar molecules, and essentially captures their chemical properties. The analytical cycle time is estimated to be <1 minute per sample, amounting to a high-throughput capacity of >4000 specimens per day, enabling the full cohort metabolome analysis (Figure S7 in the **Online Supplementary Document**). There will be rigorous quality control from the rapid liquid chromatography-mass spectrometry function to data quality and sample integrity [51-53]. This pipeline allows identification of approximately 1000 known markers and measurement of >10 000 unknown/ novel molecules. Through comparative analysis and matching to existing metabolite libraries, the structure of compounds can be derived. Once the identity of the potential metabolic signatures linked to various pregnancy complications and adverse outcomes has been confirmed, validation will be conducted in the GARBH-INi cohort.

To understand the impact of nutrition in pregnancy on maternal, foetal, and newborn health, the Consortium aims to determine the prevalence of micronutrient deficiencies among pregnant women across different geographic sites; evaluate the associations of nutrients (single and combination of nutrients) with APOs; assess the temporal changes in maternal nutritional status during the course of pregnancy; assess the relationships between maternal and foetal (cord blood) nutritional status; evaluate interactions between maternal nutritional status, inflammation, and other omics; and understand the mechanisms by which micronutrients influence human pregnancy outcomes.

Due to the high cost-per-sample and medium/low sample throughput, a case-cohort study design categorised into the sub-cohort and nested case control study (**Figure 3**) was selected. The sub-cohort includes the longitudinal samples of approximately 30% of pregnant women in the full cohort and available cord blood samples for group I nutrient analysis (Figure S8 in the **Online Supplementary Document**). Nutrients of this group include vitamin A, D, E, folate, B12, total homocysteine, ferritin, transferrin receptor, iodine, minerals (copper, selenium, magnesium, calcium, phosphorus, iron, potassium, sodium), alpha-1-acid-glycoprotein, C-reactive protein, high-density lipoprotein, low-density lipoprotein, and triglycerides. It is noteworthy that the Group I Nutrient analyses will be performed by the icddr,b, representing the only in-country analysis. Group II nutrients (vitamins B1, B2, B3, B5, B6, B7, choline, and a targeted metabolomics panel) will be analysed in the longitudinal samples of PE, SB, and vPTB cases and their matched term controls (1:3 ratio). The B vitamins, choline and related metabolites will be measured by ultra-high performance liquid chromatography (UHPLC)- MS/MS and fatty acids and amino acids by UHPLC-MS/MS – MxP®Quant 500 XL kit [51,54].

As specimens of the GARBH-INi cohort will be analysed by the Translational Health Science and Technology Institute, India (THSTI), a harmonisation plan between THSTI and icddr,b, or between THSTI and UC Davis for nutrient group I or group II analyses, respectively, was developed. Institutions will apply standardised test methods, instrument calibration, quality control measures, external quality assurance schemes, and reporting methods. The joint analysis of the nutrient group I and II parameters from the GAPPS, AMANHI and GARBH-INi sites will allow for validation across sites and analytical platforms.

The placenta tissue analysis will be particularly valuable towards the goal of designing novel diagnostics or intervention strategies to prevent APOs, as several of the APOs are either entirely or partially attributable to placental dysfunction. These include early-onset PE, foetal growth restriction, and a subset of PTB cases. Since the placenta is in direct contact with maternal blood, placental APO signatures will likely be highly complementary to APO signatures in maternal blood and provide crucial information on the involvement of certain biological pathways.

The placental tissue analysis will be limited to a smaller sample subset (as working with this tissue is labour intensive and impossible to scale-up) and focused on PTB as a common APO at all sites. Placental tissues will be profiled in a 1:2 case-cohort study by histopathology (154 PTB *vs.* 323 TB), bulk transcriptomics (26 PTB *vs.* 56 TB), single nuclei RNA and ATAC sequencing (15 PTB *vs.* 30 TB) and global proteomics by laser capture/ MS (32 PTB *vs.* 62 TB). Only one biopsy was available for the morphological analyses. Due to regional variations in placental microanatomy and based on preliminary data from the pilot study, a single endpoint (inflammation) is being evaluated [23,55]. The endpoints of the other technologies are identification of differentially expressed placental genes and proteins in placenta tissue of women with PTB *vs.* TB. The candidate biomarkers will be verified by comparisons with public databases and equivalent data from the GARBH-INi cohort.

## DATA ANALYSIS AND DATA INTEGRATION

The analytical objectives of MOMI are to determine the omics signatures of each week of advancing GA (*i.e.* temporal pregnancy progression), the strength of association of each omics or other analytical platform with each APO through machine learning modelling (thereby identifying unique features of each APO phenotype), and how site-specific effects can be used to inform model generalisation. Analysis will proceed systematically from using risk signatures derived from a single platform to signatures identified through integration of multiple omics risk signatures, and from individual cohorts to cross-cohort integration. The integration of phenotypic metadata, including sociodemographics and clinical variables, will further enhance the understanding of biological mechanisms underlying APOs and improve prediction of APOs.

The structural heterogeneity across global cohorts presents a central analytical challenge that will be addressed by explicit methodological safeguards against batch effects, missingness, site heterogeneity, and overfitting. The MOMI analytical framework is built on a mature and extensively validated computational pipeline developed and applied across multiple large, multi-site pregnancy cohorts, including prior MOMI studies [17,19] and in subsequent multi-omic integration and machine-learning frameworks [15,18,20,21]. In these works, we have implemented harmonised preprocessing and quality control, explicit modelling of batch and site effects, and integration strategies that preserve biological signal while mitigating technical variation (*e.g.* empirical Bayes and factor-based normalisation, network-based integration, and multi-view latent variable models). Missing data are handled through a combination of modality-aware imputation and model architectures that accommodate partially observed samples, enabling inclusion of participants with incomplete data without introducing bias.

For non-machine learning association analyses, we will use a prespecified covariate framework grounded in clinical relevance and established epidemiologic confounders, including maternal age, body mass index, parity, GA at sampling, and study site. These variables will be incorporated within mixed-effects or hierarchical regression models to appropriately account for cohort structure and site-level heterogeneity, with model specifications defined *a prior* to avoid data-driven overfitting. To control for false-positive findings across the large number of molecular features tested, we will apply false discovery rate correction within each omics layer and, where appropriate, across integrated analyses.

Although statistical harmonisation approaches such as ComBat are effective at reducing batch effects, they may inadvertently remove meaningful biological or population-level differences between sites. Therefore, our primary framework prioritises external validation across sites as the most conservative test of reproducibility and transportability. Rather than relying solely on post hoc harmonisation, our primary strategy is to evaluate robustness and generalisability directly. Specifically, we will train models on subsets of study sites and validate them on held-out sites. This design allows us to assess real-world performance under true site-level variation, including differences in GA assessment, biospecimen handling, and sociodemographic context. This cross-site training and validation framework ensures that identified signatures are not artifacts of a single cohort, platform, or operational pipeline, but instead reflect patterns that are stable across diverse populations. Only models and associations that replicate across sites will be interpreted as biologically meaningful, providing a principled basis for pooled analyses. As a complementary, secondary analysis, we will apply ComBat-based correction and mixed-effects modelling to quantify and compare the impact of site-level harmonisation. This layered approach allows us to rigorously test whether pooled analyses are justified, distinguish technical from biological sources of heterogeneity, and preserve interpretability, while maintaining statistical rigor across structurally heterogeneous cohorts.

The computational pipeline employs dimensionality reduction and unsupervised clustering for sample classification, followed by statistical modelling to identify signatures associated with adverse outcomes while accounting for confounding factors. Longitudinal analysis characterises trajectories during normal pregnancy progression, providing essential context for understanding pathological changes. In parallel to the statistical approaches, machine learning will be applied to identify signatures with predictive value to enable the development of assays with diagnostic and prognostic value for the identification of APOs. This multi-layered analytical strategy aims to distinguish universal molecular signatures from population-specific variations. Pathway enrichment analysis is an essential step for interpreting the results of predictive models in biological terms, particularly when the aim is to understand the molecular mechanisms of distinct APOs. The different omics platforms will employ distinct pathway analysis tools, such as KEGG Pathway Database, Gene Ontology, STRING, MetaboAnalyst, and others [56-58]. Pathway enrichment will be conducted separately for each site and APO to ensure that the localised biological processes are accurately captured. To predict APOs of interest, machine learning techniques, specifically models based on eXtreme Gradient Boosting [59], a powerful and efficient implementation of gradient boosted decision trees, will be used. For each outcome, a distinct model will be constructed at various pregnancy stages to capture the static risk at that time point. One hundred bootstrap iterations will be performed during which a subset of the patients equal to the size of the full data set will be selected randomly with replacement. This method involves repeated random sampling to split the data into training and validation sets, thus providing a thorough assessment of model performance across various data subsets.

To control for site imbalance in a multi-country cohort, all resampling procedures will be performed using stratified bootstrapping that preserves the joint distribution of site and outcome status. Specifically, bootstrap samples will be drawn within strata defined by study site and outcome (and, where relevant, by case-cohort membership), ensuring that each resample reflects the original cohort’s structural heterogeneity. This approach maintains appropriate representation of under-represented sites and rare outcomes in each iteration, preventing dominance by large sites and reducing bias in variance estimation and model stability assessments. Stratified resampling will be used consistently for uncertainty estimation, feature stability analyses, and performance evaluation, providing a more rigorous assessment of robustness in the presence of site imbalance. The GARBH-INi data will be utilised to validate data generated by the MOMI cohorts and, *vice versa*, GARBH-INi findings may be validated using the MOMI cohort data. The performance of these predictive models will be evaluated using area under the receiver operating characteristic and the area under the precision-recall curve, as well as prevalence of the case. Multi-site analysis is a critical component of model building, ensuring that the predictive models are robust and applicable across different populations and conditions.

A major goal of the MOMI analysis is to move beyond purely associative multi-omics analyses toward mechanistic insight into pathways driving APOs. Thus, we will apply causal inference methods to identify key pathways leading to APOs and inform strategies on their modulation to prevent APOs. Existing causal and footprint-based frameworks (*e.g.* Causal Oriented Search for Multi-Omic Space) were developed primarily in oncology and rely on prior knowledge networks that are sparse for pregnancy biology, omit key pregnancy-specific molecules (*e.g.* pregnancy-specific glycoproteins), and do not natively integrate genomics with multi-omics in this context. We therefore developed Adverse Pregnancy Outcome Learning through a causaL-oriented multi-Omic (APOLLO) search, a pregnancy-aware causal network framework that integrates differential multi-omic signals with curated pregnancy-specific prior knowledge, augmented by data-driven and literature-derived edges. The APOLLO will operate under standard causal inference assumptions (temporal ordering, no unmeasured confounding within modelled graphs, and consistency) and will explicitly encode directionality of regulation. It will be validated by applying it across APOs in the MOMI Consortium and benchmarking its inferred pathways against COSMOS and expert-curated analyses, ensuring that resulting networks are biologically grounded, pregnancy-relevant, and reproducible across sites.

Our planned MR analyses will be guided by a biologically motivated causal framework in which genetic variation precedes molecular variation, which in turn influences pregnancy outcomes. This framework is well supported by prior literature demonstrating causal relationships between genetically regulated trait, such as height, BP, glucose metabolism, lipid levels, and inflammatory processes, and pregnancy outcomes. Where MR is applied, instruments will be selected using established criteria, such as strong association with the exposure (genome-wide significance), independence from confounders, and absence of horizontal pleiotropy (assessed via sensitivity analyses such as MR-Egger and leave-one-out tests). Genetic instruments for molecular traits will be restricted to cis-acting variants where possible to enhance biological interpretability. Together, the MOMI study cohort design and analytical approaches ensure statistical efficiency, cost-effectiveness, and unbiased inference, while enabling robust discovery and validation of molecular signatures across rare and common pregnancy outcomes.

## DISCUSSION

In the last two decades, we have achieved a 34% reduction in maternal mortality and a 44% decrease in neonatal deaths. Yet globally, maternal and infant mortality remain unacceptable high, with 223 deaths per 100 000 live births occurring in 2020 and 2.3 million children dying within the first 28 days of life in 2023 [32]. Pregnancy complications and APOs are a major contributing factor in these deaths.

The MOMI Consortium is performing a multi-omics analysis of unprecedented scale across highly diverse populations of LMICs in South Asia and Africa to identify diagnostic markers for early detection of pregnancy complications with the goal to reduce maternal and neonatal mortality and to improve long-term maternal and infant health outcomes. The only other study of similar scale in cohort size, that we are aware of, is currently ongoing in China [4].

A significant milestone of the MOMI project was the harmonisation of study protocols across sites which encompassed study design and sampling methods, adjudication for the key APOs, as well as continued development and optimisation of omics platforms and analysis tools across the partner sites. Adherence to these standardised protocols by all collaborators ensures consistency and quality control in data collection, downstream analysis, and the generation of biologically meaningful insights that are comparable across the sites. Although chronic hypertension could not be systematically excluded at enrolment across all sites, the Consortium applied a conservative, multi-tiered adjudication framework to preserve PE phenotype specificity. Consequently, high-confidence PE cases are unlikely to represent isolated chronic hypertension, and any residual misclassification is expected to bias associations toward the null rather than generate spurious positive findings.

The probing of longitudinal maternal samples with cord blood and placental tissue at delivery by multiple distinct analytical platforms and the subsequent inter-omics data integration will provide a comprehensive view on normal temporal changes of biological pathways in pregnancies progressing to term and birth of a live infant at appropriate weight *vs.* pregnancies resulting in APOs. While each analytical data set may provide insights into one or more APOs, the power of the study lies in the data integration across multiple platforms that will enable a deeper pathway interrogation and increase confidence into signals associated with APOs. Furthermore, the comparative analysis of data across the various sites will reveal site-specific *vs.* common factors that underlie specific APOs. Site-specific variables, such as genetic background, diet, socio-economic factors, or HIV infection are likely to impact pregnancy outcomes of women in their respective cohorts. Nonetheless, we anticipate identifying signatures common to women with the same APO and specific for that APO. The latter signatures will be most valuable for earlier diagnostics and precision interventions that can improve pregnancy care and outcomes globally. Simultaneously, care can be further improved through additional and complementary site-specific interventions. The data obtained from the placenta analysis are expected to provide novel insights into maternal-foetal interactions and, in combination with the data from the infant follow-up visits, inform about the link between placental function and long-term health risks in offspring. Such knowledge will inform implementation of post-partum interventions to ameliorate long-term health consequences for the infant.

The MOMI initiative represents a landmark study and is expected to serve as a roadmap for future investigations relevant to maternal, foetal, and infant health and other disease-related programmes. The principal investigators at each site will share their main findings with scientific communities and local governmental law- and decision-making institutions to pave the way for policy and decision-making processes that can lead to nationwide interventions and influence clinical practices across the country. The combined gain in knowledge and its conversion to actionable diagnostics and therapeutics is expected to have significant impact on the lives of pregnant women beyond LMICs. The inclusion of women from five different countries on two continents, living in rural or urban areas, in the current study is unprecedented and the results will generate insights into pregnancy progression and APOs that is likely to transform the lives of women and infants worldwide, extending its influence beyond the immediate participants.

To summarise, the MOMI study utilises a multi-disciplinary approach towards making informed choices for diagnostic and therapeutic product development. Accordingly, the knowledge gained from this study in terms of identification of reliable and generalisable targetable biomarkers as well as in-depth knowledge on the precise pathogenesis and associated drivers will drive global translational and transformational change in clinical practices on a global scale.

## Additional material


Online Supplementary Document
