# Supplementary material for: Multi-Omics for Mothers and Infants (MOMI) Consortium: a global initiative to study adverse pregnancy outcomes
Source: J Glob Health. 2026 Apr 3;16:05002. doi: 10.7189/jogh.16.05002 (PMC13045933; doi:10.7189/jogh.16.05002)
Supplement: Online Supplementary Document [file jogh-16-05002-s001.pdf]

Supplement to:Tang X, Ahmed S, Ahmed FB, Albrecht V, Brennsteiner V, Chang AL, Chowdhury NH, Deb S, Gormley M, Hasan T, Kamulaza L, Khalid J, Khan W, Khanam R, Kshetrapal P, Luo H, Maiti TK, Maitra A, Mannan MM, Mueller-Reif J, Mulenga C, Nisar I, Pervin J, Pokaprakarn T, Price J, Qazi MF, Rittenhouse K, Sebastiao Y, Sindano N, Sinha T, Sopory S, Steger M, Thiruvengadam R, Wadhwa N, Xue L, Aghaeepour N, Baqui AH, Bhatnager S, Fisher S, Fox M, Hampel D, Kasaro M, Mann M, Rahman A, Sazawal S, Shenhav L, Stringer JSA, Zhang G, Jehan F, De Paris K. Multi-Omics for Mothers and Infants (MOMI) consortium: a global initiative to study adverse pregnancy outcomes. J Glob Health. 2026;16:03008.

Table S1. Number of specimens included in the multi-omics analysis

Figure S1. MOMI Consortium organizational structure

Figure S2. Stillbirth decision tree

Figure S3. Preeclampsia decision tree

Figure S4. Genomics analysis

Figure S5. CpG methylation analysis

Figure S6. Proteomics workflow

Figure S7. Metabolomics analysis

Figure S8. Selection of women for nutrient Group 1 and Group 2 analyses

Table S1.Number of Specimens included in Multi-Omics Analysis

Sample Type and Timepoint per Analysis	Specimen Number/ Site					
	GAPSS		AMANHI			MOMI Consortium
	Zambia	Bangladesh	Bangladesh	Pakistan	Tanzania	
**Genomics**						
Maternal DNA[a]	1,442	3,567	1,803	2,004	3,813	*12,629*
Cord Blood DNA	439	2,027	1,495	1,491	3,377	8,829
**Epigenomics**						
Maternal Blood[a]	235	501	193	261	530	*1,720*
Cord Blood	87	414	150	158	363	1,172
**Metabolomics**						
Maternal Plasma						
<20 wks GA	1,936	3,441	2,670	2,481	4,183	14,711
20-24 wks GA	325	2,643	234	304	4	3,510
24-28 wks GA	1,680	369	1,764	1,638	1,817	7,268
28-32 wks GA	766	1,323	194	36	43	2,364
32-36 wks GA	832	1,499	378	245	1,970	4,924
>36 wks GA	5	1	3	0	14	23
						Total: 32,800
Cord Blood Plasma	1,167	2,288	294	1,851	7,045	12,645
**Proteomics**						
Maternal Plasma						
<20 wks GA	1,478	3,795	2,676	2,480	4,484	14,913
20-24 wks GA	409	2,793	391	649	73	4,315
24-28 wks GA	1,396	266	1,623	1,287	1,856	6,428
28-32 wks GA	565	1,600	207	65	168	2,605
32-36 wks GA	449	1322	345	174	1,983	4,273
>36 wks GA	6	1	14	1	5,917	5,939
						Total: 38,473
Cord Blood Plasma	429	2,278	1,441	292	3548	7,988
**Nutrition I**						
Maternal Serum						
<20 wks GA	718	1,042	1,016	1,037	1,106	4,919
20-24 wks GA	27	823	75	134	12	1,071
24-28 wks GA	259	92	682	706	467	2,206
28-32 wks GA	545	46	82	16	37	726
32-36 wks GA	0	52	124	102	526	804
>36 wks GA	0	0	5	4	24	33
						Total: 9,759
Cord Blood Plasma	480	844	814	155	1,040	3,333
Maternal Urine						
<20 wks GA	701	1,034	1,016	1,037	1,096	4,884
20-24 wks GA	0	872	76	134	12	1,094
24-28 wks GA	152	100	685	706	467	2,110
28-32 wks GA	528	24	82	16	37	687
32-36 wks GA	0	30	125	102	526	783
>36 wks GA	0	1	5	4	24	34
						Total: 9,592

**Nutrition II**						
Maternal Plasma[a]	124	256	185	240	447	*1,252*
Cord Blood Plasma	78	196	138	34	412	858

[a] only a single time point (<24 wks GA)

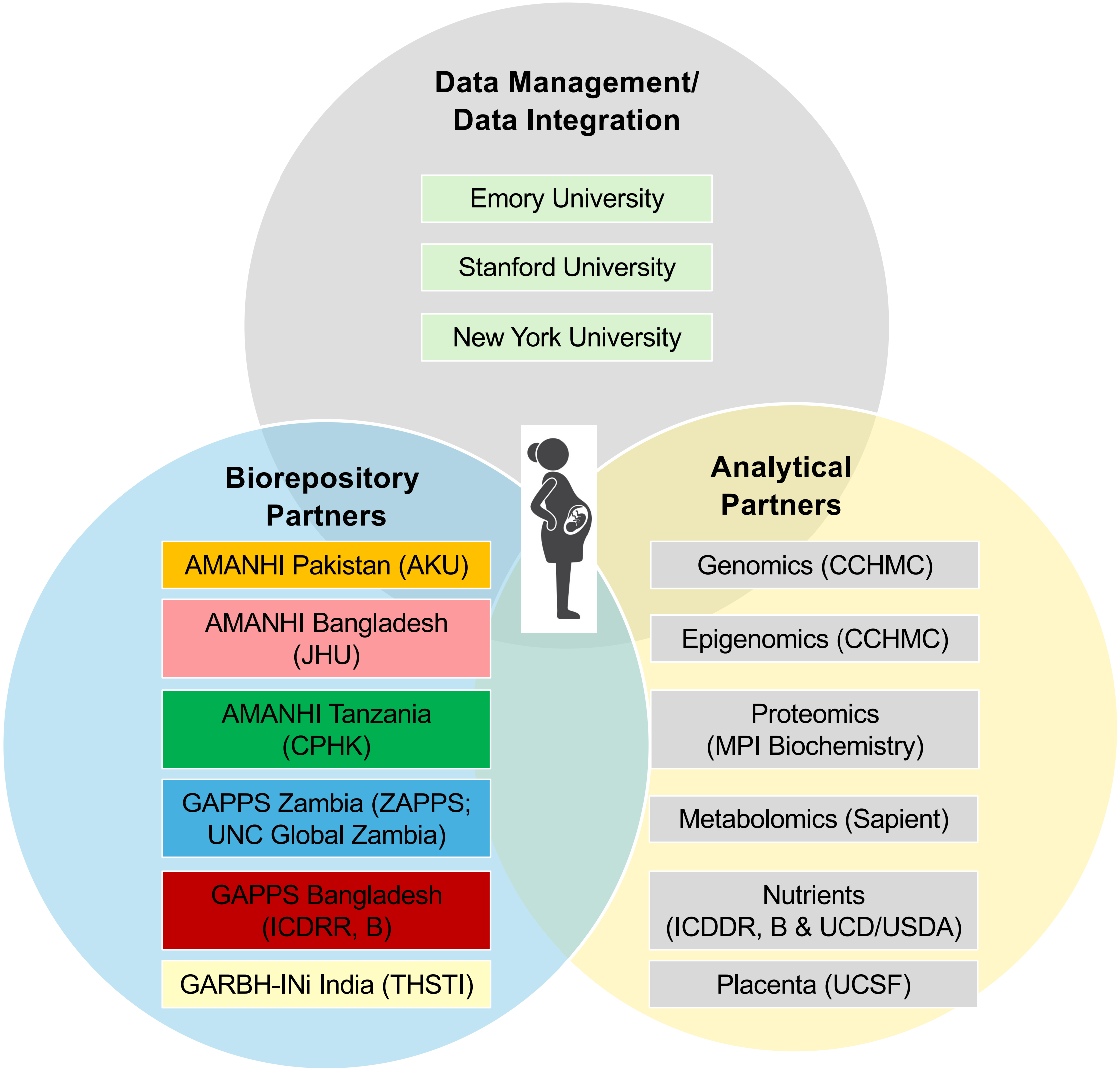

Data Management/
Data Integration
Emory University
Stanford University
New York University
Biorepository
Partners
AMANHI Pakistan (AKU)
AMANHI Bangladesh
(JHU)
AMANHI Tanzania
(CPHK)
GAPPS Zambia (ZAPPS;
UNC Global Zambia)
GAPPS Bangladesh
(ICDRR, B)
GARBH-INi India (THSTI)
Analytical
Partners
Genomics (CCHMC)
Epigenomics (CCHMC)
Proteomics
(MPI Biochemistry)
Metabolomics (Sapient)
Nutrients
(ICDDR, B & UCD/USDA)
Placenta (UCSF)

Figure S1: MOMI Consortium Organizational Structure. The MOMI Consortium consists of three main arms, The Biorepository, Analytical, and Data Management partners. Investigators from all components work closely together in a highly collaborative way to define pathways of normal pregnancy with live term birth and identify biological mechanisms associated with adverse pregnancy outcomes.*The GARBH-INI site will perform its own analytical analyses, as the site has no permission to ship specimens out of the country. Therefore, the GARBH-INI cohort is separate from the MOMI cohort. Nonetheless, sample analytics, as well as data analysis and integration are coordinated with the MOMI cohort.

Abbreviations: AKU = Aga Khan university, Pakistan; JHU = John Hopkins University, USA; CPHK = Center for Public Health Kinetics, Tanzania; UNC = University of North Carolina at Chapel Hill, USA; ICDDR,B = International Centre for Diarrhoeal Disease Research, Bangladesh; THSTI = Translational Health Science and Technology Institute, India; CCHMC = Cincinatti Children's Hospital Medical Center, USA; MPI = Max Planck Institute of Biochemistry in Munich, Germany; Sapient = Sapient Bioanalytic LLC, San Diego, USA; USDA/ UCD = United States Department of Agriculture/ University of California at Davis, USA.

Figure S2: Stillbirth decision tree

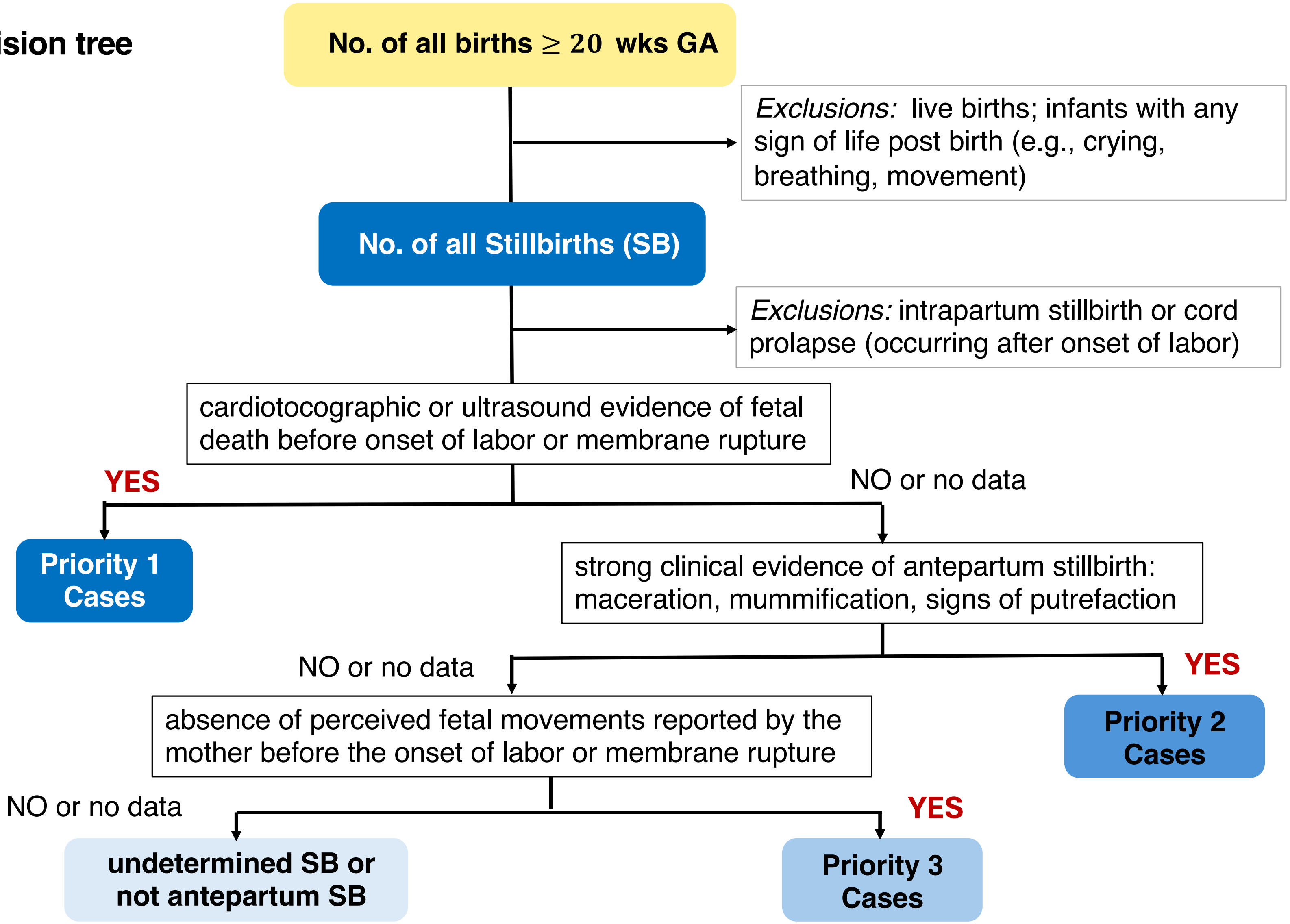

Figure S3: Preeclampsia decision tree

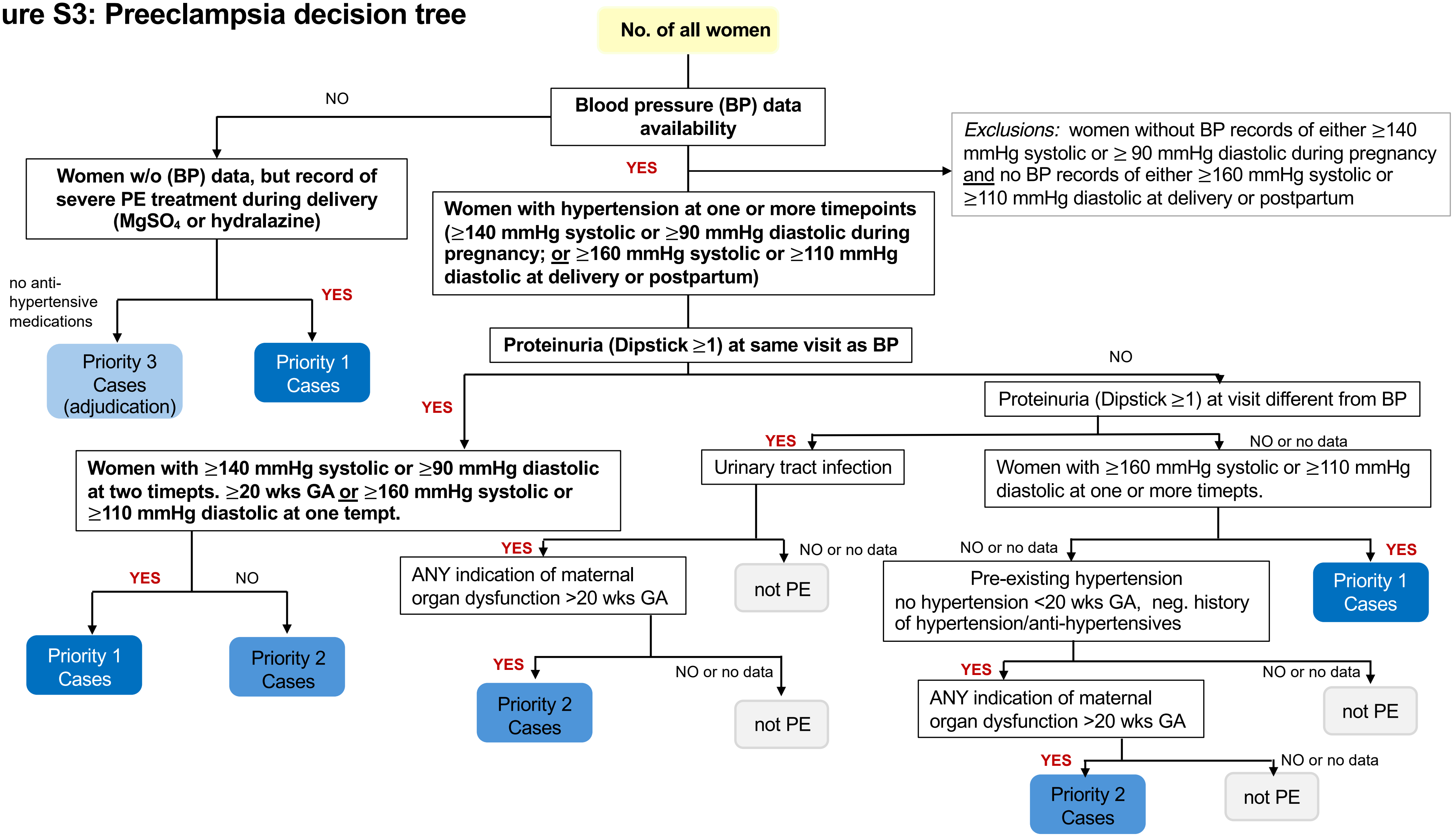

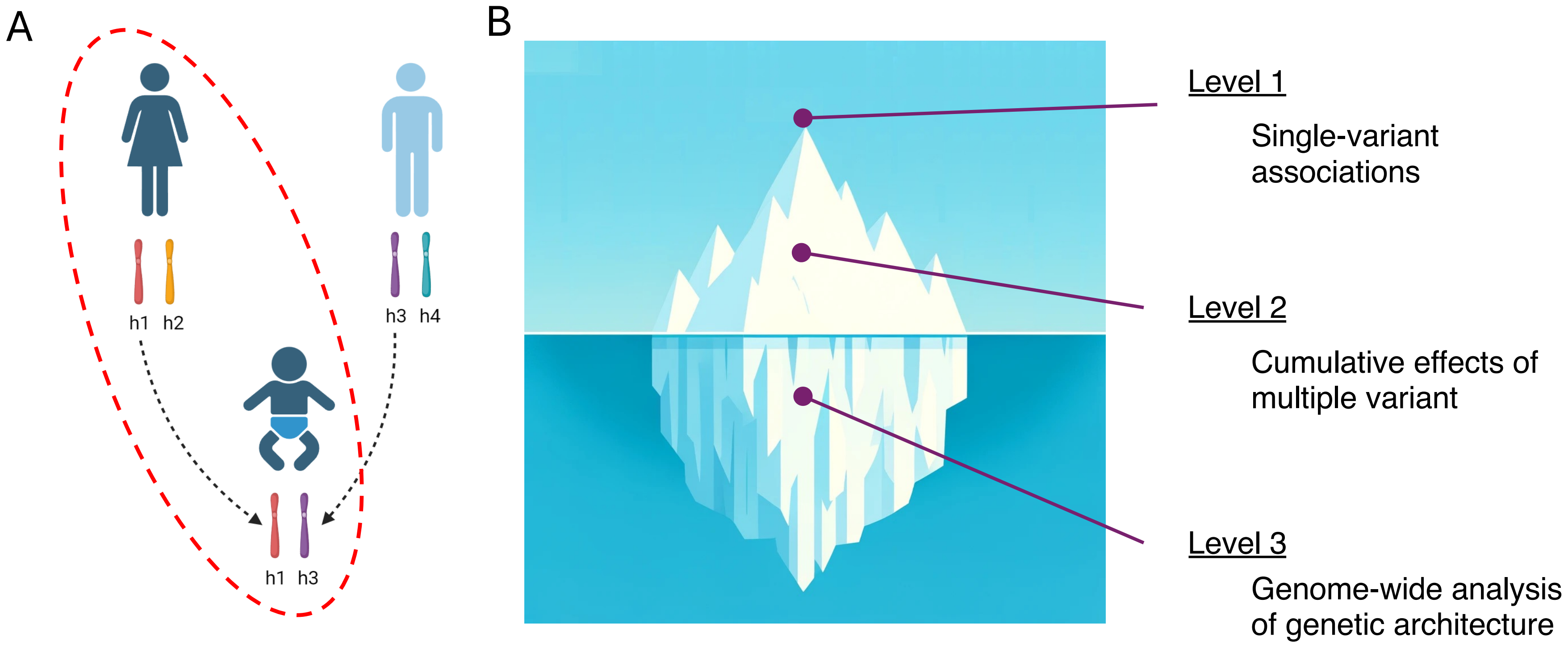

Figure S4: Genomics Analysis. *Panel A:* Haplotype-based genomic analysis using mother/child duos as an "analytical unit". The maternal and fetal genetic effects can be dissected by jointly testing the maternal transmitted alleles (h1, with both maternal and fetal effect), the maternal non-transmitted alleles (h2, maternal effect only) and the paternal transmitted alleles (h3, fetal genetic effect only). The three haplotypes (h1,h2,h3) in one pregnancy are included in red dash circle. *Panel B:* Genomic data analysis at three different levels: level 1) single-variant analysis to replicate known genetic associations and identify novel population-specific or enrich associations; level 2) multi-variant analysis to test various polygenic scores and their association with pregnancy outcomes; level 3) the overall maternal and fetal genetic contributions and their correlations in shaping pregnancy outcomes .

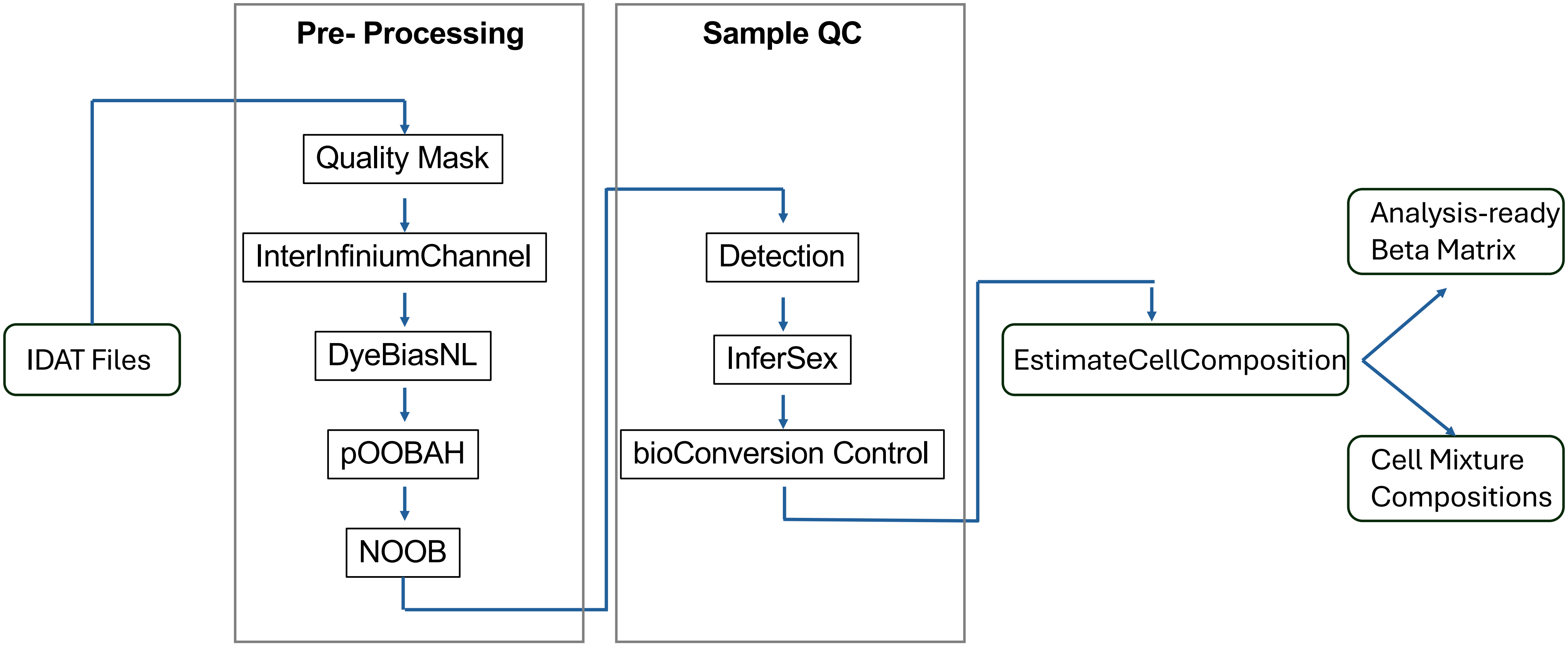

Figure S5: CpG Methylation Analysis. The workflow of methylation array data processing will target over 935,000 CpG sites in biologically significant regions. R package “SeSAME” will be used to pre-process and QC the data. Each box indicates the R function of the corresponding step. The input is the raw data IDAT files, and output are the analysis-ready beta-value matrix and cell composition matrix for all samples.

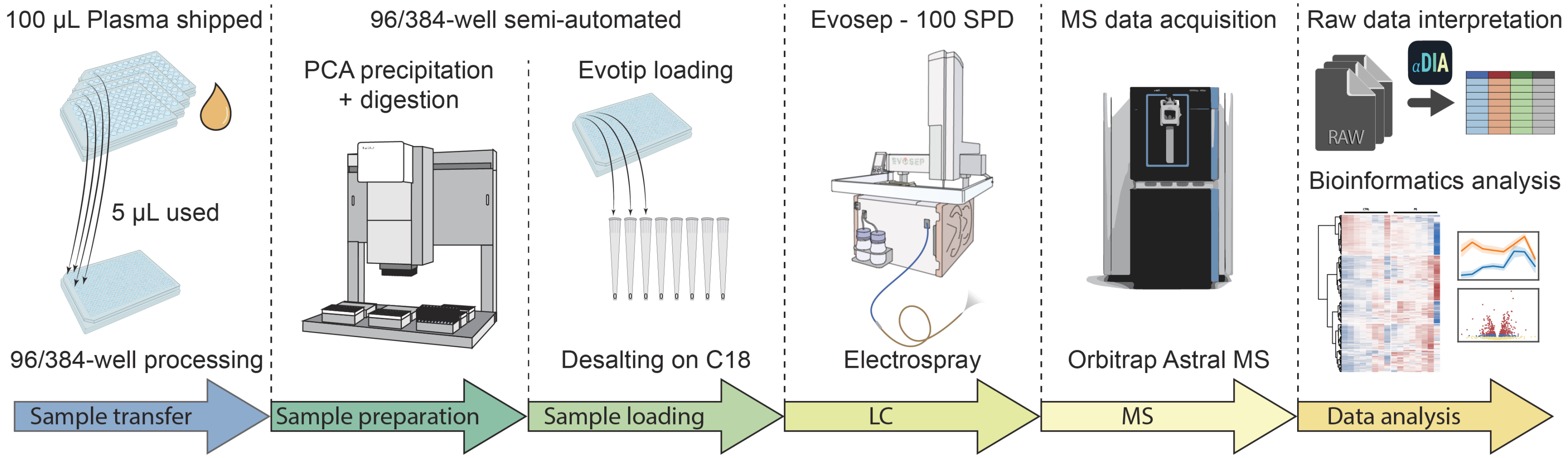

Figure S6: Proteomics Workflow. Schematic workflow of plasma proteomics analysis from sample processing to labeling and LC/MS analysis.

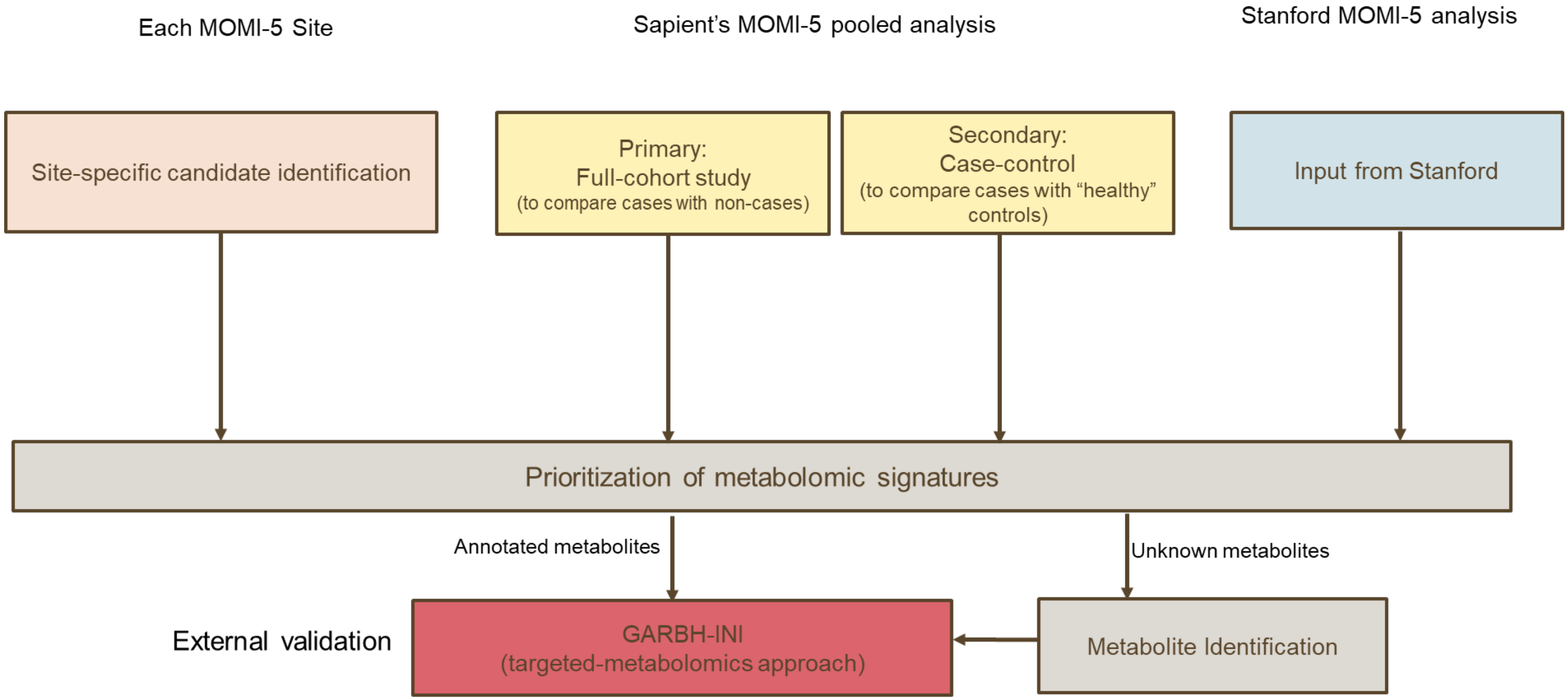

Figure S7: Metabolomics Analysis. Each of the GAPPS and AMANHI cohorts will perform a site-specific analysis. Data of the full MOMI cohort will undergo a primary analysis comparing all term births versus all cases (APOs) and a secondary, smaller case:control study. The case:control study will focus on a unique APO and compare metabolomic signatures to those obtained from matched women with term birth and healthy infant. The Stanford data analysis team will assist in data validation and application of machine learning approaches to identify APO-specific metabolites. Those will then be validated by comparing them to plasma metabolome signatures of women with the same birth outcome in the GARBH-INI cohort.

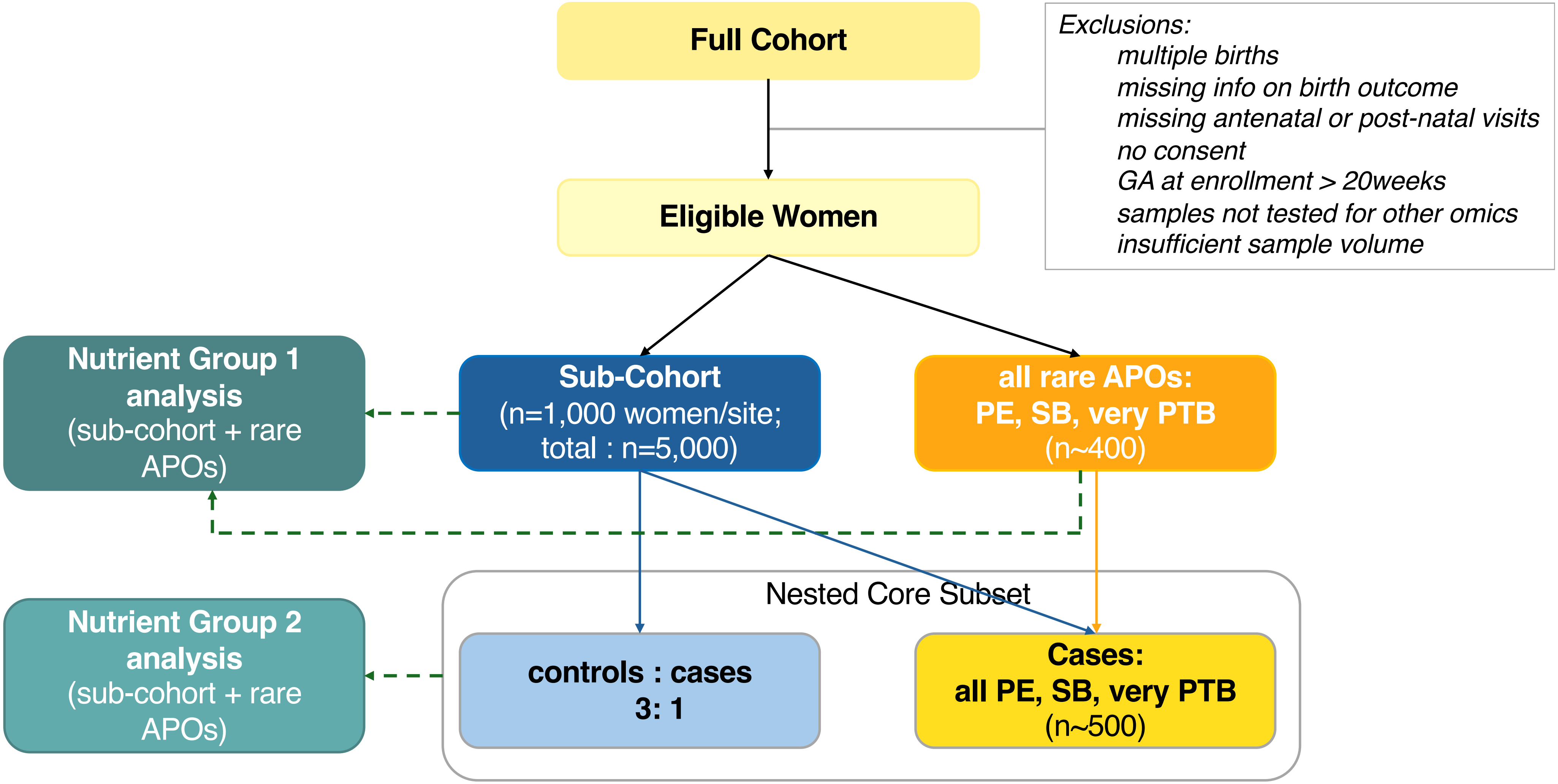

Figure S8: Selection of women for Nutrient Group 1 and Group 2 analyses.
